# Our Thanks

**Published:** 1872-12

**Authors:** 


### OUR THANKS.

We sincerely thank those of our friends who have suggested a new name for the journal, in accordance with our request. The new name will be given the journal January next, and we trust it will be more satisfactory, than the old, to all our readers and friends.

We also thank those of our friends—a large number—who have sent us new subscribers for the next volume, and who propose to secure from three to five subscribers in accordance with the request of one of our Associates, published in the November number. We hope *all* will consent to aid in the circulation of the journal upon this plan, among their friends. Every subscriber has his friends and but a little personal effort will be required to secure their subscriptions.

We also beg to remind our friends to send the journal the results of their experiences as practitioners. Report your cases. Many valuable facts are lost to medical literature because practitioners do not esteem their cases—many of them—important enough for publication. We beg them to publish their cases, and freely offer them the use of our columns for that purpose. Will they not give heed to this request? We hope so.

